# Pleomorphic Giant Cell Carcinoma and Periurethral Abscess: A Case Report

**DOI:** 10.7759/cureus.72533

**Published:** 2024-10-28

**Authors:** Farhan Jarral, Guleed Mohamed, Anand Mohan, Rosa Mobayen, Jakub Wrazen, Osama Abusand, Veena Naik, Francis Thomas, Ramanan Rajasundaram

**Affiliations:** 1 Urology, Doncaster Royal Infirmary, Doncaster, GBR; 2 Urology, Bradford Royal Infirmary, Bradford, GBR; 3 General Surgery, Ealing Hospital, London North West University Healthcare NHS Trust, London, GBR; 4 Urology, Chesterfield Royal Hospital, Chesterfield, GBR; 5 Histopathology, Sheffield Teaching Hospitals NHS Foundation Trust, Sheffield, GBR

**Keywords:** oncology, pathology, sarcomatoid carcinoma, urethral disease, urological cancer

## Abstract

Prostate cancer (PCa) is the most common solid malignancy in men in the UK. Pleomorphic giant cell carcinoma (PGCC) is a rare, aggressive variant of prostate adenocarcinoma. PGCC is associated with a poor prognosis and high Gleason-grade characteristics, often occurring in patients with a history of PCa treatment. This case report details the presentation of a 78-year-old male with a background of PCa, previously treated with radiotherapy and androgen deprivation therapy, who was initially diagnosed with a periurethral abscess. Despite initial treatment, the patient experienced recurrent symptoms, leading to further investigations and surgical intervention. Histopathological analysis of tissue samples revealed PGCC, highlighting the importance of considering this malignancy in cases of recurrent abscesses in patients with a history of PCa. This case underscores the necessity of early suspicion, prompt investigation, and multidisciplinary management in complex cases involving PGCC, emphasizing the need for heightened awareness of this rare pathology in clinical practice.

## Introduction

Prostate cancer (PCa) is the most common solid malignancy in men in the UK [1]. Prostatic neoplasms consist of a wide array of histological variants. One recognized, although extremely rare, histological subtype of acinar adenocarcinoma of the prostate is pleomorphic giant cell carcinoma (PGCC) [2]. PGCC is known to have an aggressive pathology with a poor prognosis despite treatment. PGCC frequently exhibits high Gleason grade characteristics. It is currently placed in The International Society of Urological Pathology (ISUP) grade group 5, indicative of the most aggressive and very high risk of spread [3]. PGCC has been reported in patients with PCa who have received previous treatment such as androgen deprivation therapy, chemotherapy, and radiotherapy [4].

Most patients diagnosed with PGCC present with locally advanced disease or metastatic disease and thus may only be suitable for palliative treatment [5].

PGCC has also been found to exist in other organs such as the lungs, urinary bladder, and pancreas, which therefore makes diagnosis of the primary organ a challenge [6]. This, therefore, requires further immunohistochemical investigations to identify the primary origin [7]. Early and prompt recognition of the tumor is essential for planning and initiating aggressive treatment.

We present the case of a 78-year-old gentleman with a background of PCa who presented to the accident and emergency department with what was deemed a scrotal abscess but was found to have PGCC on further investigation.

## Case presentation

A 78-year-old male with a background of type 2 diabetes mellitus (T2DM), hypertension, intermittent self-catheterization (ISC), and Gleason 4+5=9 prostate adenocarcinoma that was previously treated with radical radiotherapy and androgen deprivation therapy presented to the accident and emergency department with a three-day history of painful scrotal swelling with associated dysuria and fevers.

On examination, he was found to be febrile with scrotal swelling with no obvious sinister features on inspection or palpation. During the initial assessment of this patient, he had routine blood tests at the emergency department that demonstrated a white cell count of 13.8 x 10^9/L, neutrophils of 11.8 x 10^9/L, and C-reactive protein (CRP) of 290 mg/L. A urine culture was also sent on admission, which was negative for any growth.

As part of the workup for his acute scrotum, an ultrasound scan was ordered, which showed scrotal wall edema and a normal ultrasound appearance of both testes and epididymides. Following a two-day admission and being commenced on treatment for epididymo-orchitis, he was discharged home with a plan to be seen in the urology outpatient clinic in a week as his inflammatory markers and pain had improved.

The patient was seen in the urology outpatient clinic one week post-discharge as planned and felt generally unwell and began complaining of worsening scrotal pain. Physical examination revealed a tender, warm right hemi-scrotum with purulent discharge per urethra. He was promptly re-admitted from the clinic to the ward for further investigations and treatment.

A repeat ultrasound test demonstrated an 8.6 x 8.2 x 7.8 cm area of mixed echogenicity within the right hemi-scrotum, inferior to the testis in keeping with an evolving abscess. Both testes were normal ultrasonically.

**Figure 1:**
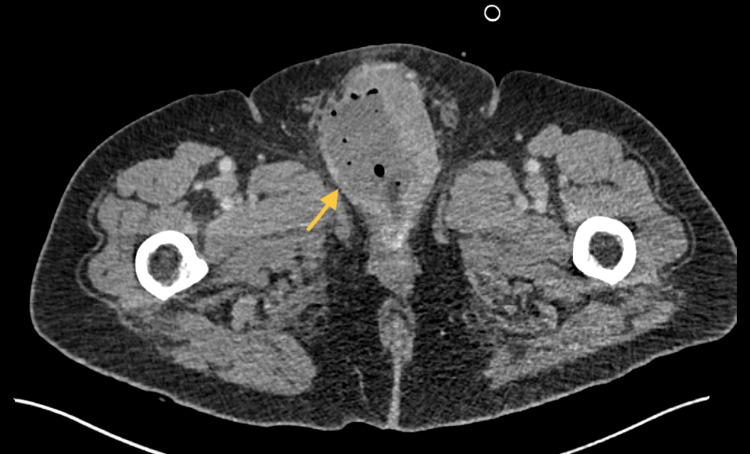
Initial CT scan demonstrating perineal inflammatory condition

CT abdomen and pelvis (CT AP) with contrast was performed and found there was a large inflammatory perineal collection involving the proximal penile shaft and extending down to the cranial aspect of the scrotum, consistent with an abscess (Figure 1).

A decision was made to insert a suprapubic catheter (SPC) and drain the periurethral abscess in the theater under general anesthesia. Examination under anesthesia found a swollen scrotum, with no area of gangrenous or necrotic patches on the skin. No perianal pathology was found. An SPC was inserted, and both sides of the urethra were dissected to connective tissue, and pus was washed out. A corrugated drain was placed to drain the perineal wound. Urine and pus samples were taken intraoperatively and sent for culture, but both were negative for growth.

**Figure 2:**
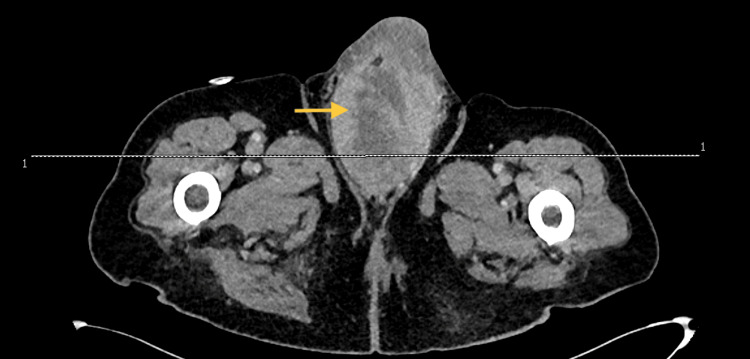
CT scan representing abnormality involving root of penis at subsequent presentation

On postoperative day five, a repeat CT AP with contrast was performed, which reassuringly showed that the perineal collection had reduced in size (Figure 2). The perineal drain was subsequently removed, and the patient was discharged home on oral antibiotics.

Unfortunately, a few days later the patient was re-admitted due to pain and noting pus discharging from the operative wound around the right side of the scrotum and the base of the penis. He underwent another CT AP with contrast, which demonstrated a large heterogenous enhancing collection with probable abscesses at the base of the penis and surrounding his urethra. While an inpatient, he was managed with antibiotics and was discharged when he became stable.

Following discharge, the patient presented to the emergency department on the same day with increased swelling and pain in his scrotum. Examination showed an enlarged left hemiscrotum and mild enlargement of the right scrotum. There were no skin changes or obvious sinister features that pointed in the direction of Fournier’s gangrene. Another CT AP was ordered, which showed the abnormality involving the root of the penis posteriorly had increased in size, now measuring 12 x 7.5 x 7 cm compared to 6 x 3.5 x 3 cm on the previous CT AP performed two months prior. There was no extension of the collection in the abdominopelvic cavity.

A decision was made to take him back to the theater for a second time, where he underwent an emergency incision and drainage of the collection. Intraoperatively, there was a large globular mass in the midline of the perineum fixed to the urethra and indenting the urethra with the communication discharging pus. The cavity was full of thick debris that was shelled out. The debris appeared malignant in nature, and the proximal urethra was completely destroyed by what appeared to be either infection or cancer. The catheter was palpable at the level of the proximal urethral within the abscess cavity. During the procedure, tissue samples were sent for histology. 

**Figure 3:**
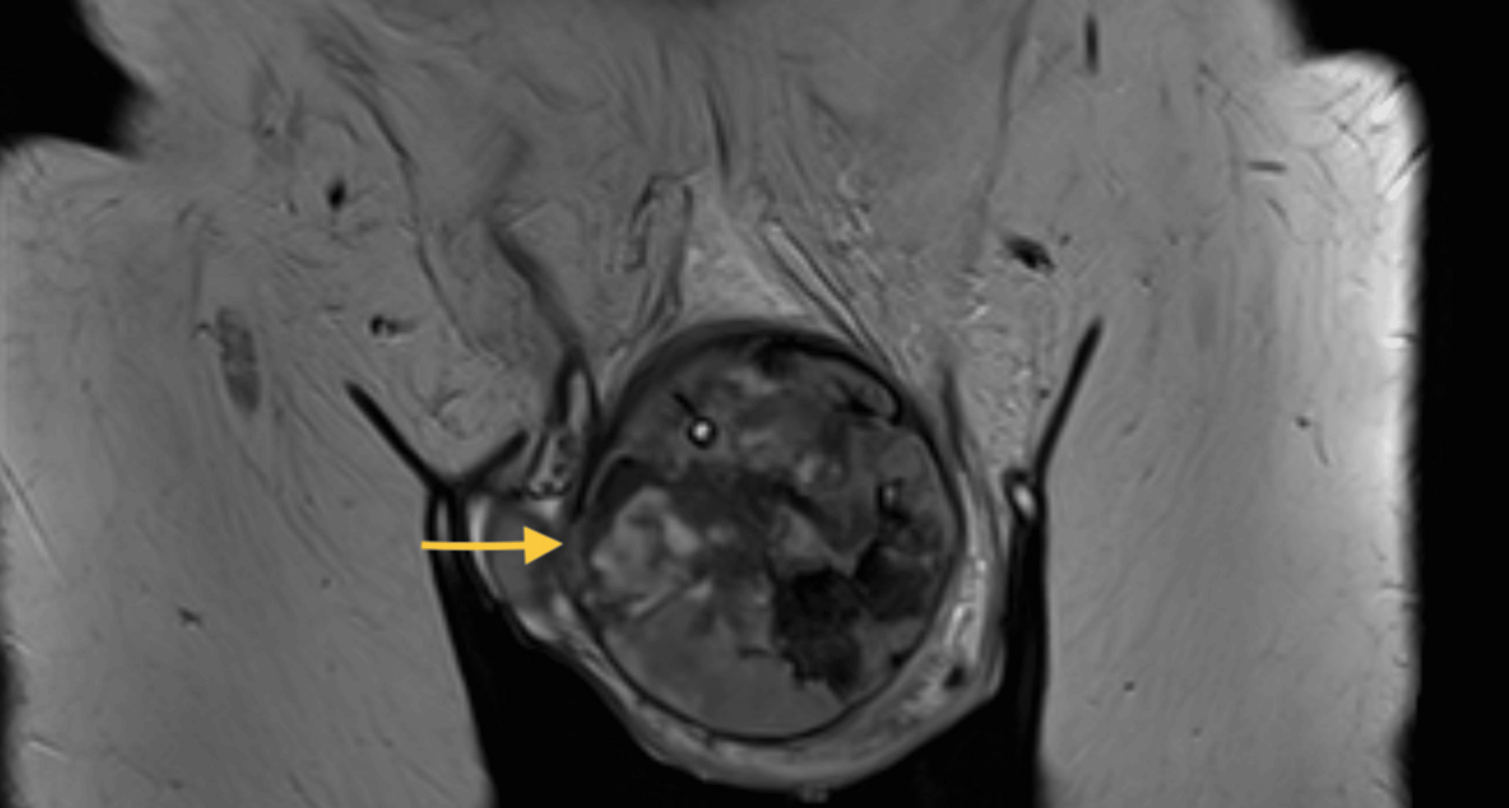
Coronal slices of MRI pelvis demonstrating malignant mass likely from penile cancer

Due to intra-operative findings, an MRI pelvis was requested to provide further information on tissue infiltration, primary or metastasis. The report showed a very large malignant mass lesion, likely primary penile cancer (Figure 3). There was no infiltration of the bones, testes, urinary bladder, or pelvic lymphadenopathy.

**Figure 4:**
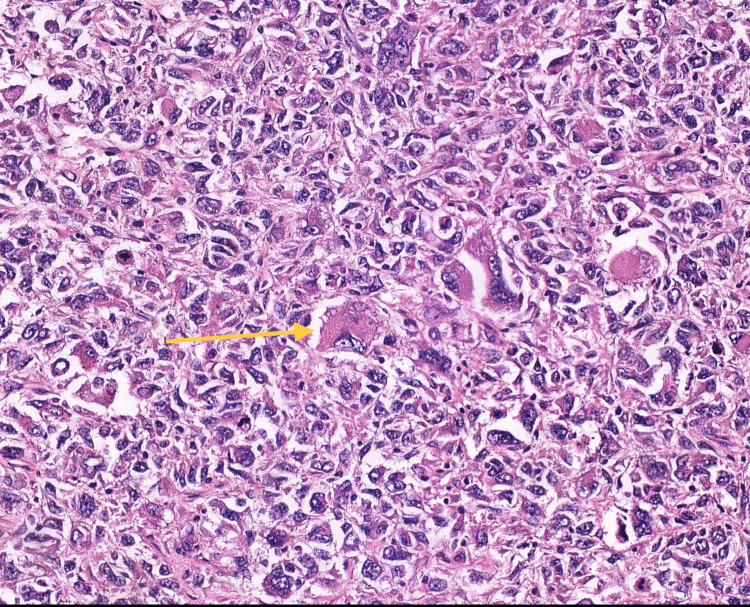
Histology: pleomorphic giant cell carcinoma. Multi-nucleated cell highlighted.

**Figure 5:**
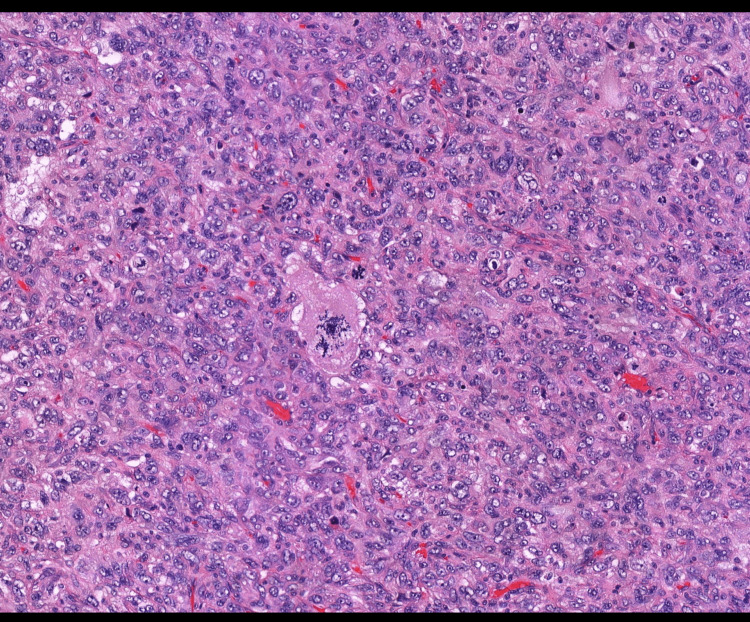
Histology: pleomorphic giant cell carcinoma

The histology report demonstrated a high-grade tumor composed of highly pleomorphic cells with multinucleate giant cells. It was diagnosed as PGCC, which is a variant of metastatic prostatic adenocarcinoma (Figures 4-5). Following a local multi-disciplinary team discussion, the patient was referred to a tertiary center for consideration of further treatment under the andrology team in conjunction with the plastics team.

## Discussion

Periurethral abscesses are rare but life-threatening infections mostly related to the peri-urethral and urethral tissues. Initially, infections may be confined to Buck's fascia. However, once this is breached, there may be extensive necrosis of fascia and subcutaneous tissue [8].

As with all clinical pathologies, the presentations may vary; however, patients with periurethral abscesses may present with dysuria, fever, urethral discharge, and swelling of the scrotum or penis [9].

Regarding risk factors, Tariq et al. reported they include sexually transmitted infection (STI)/urinary tract infection (UTI) in 55.0%, urethral strictures in 39.6%, urethral instrumentation in 18.7%, and diabetes mellitus in 6.6% whilst examining 270 cases of peri-urethral abscesses. Similarly, he found that most pathogens isolated from urine, periurethral abscess fluid, or tissue samples were anaerobic organisms. The predominant anaerobic species identified were *Escherichia coli*, *Enterococcus*, and various species of *Staphylococcus *and *Streptococcus*. In contrast, the aerobic pathogens detected included *Neisseria gonorrhoeae* and *Corynebacterium* species [10]. In this specific case report, swab and urine cultures were performed during each hospital admission, but none showed significant growth.

The patient in this case has a history of ISC as well as T2DM, both of which are significant risk factors for the development of a periurethral abscess.

Periurethral abscesses can typically be treated using either conservative or surgical approaches. Initial management can involve placing an SPC to divert urine, which was done in this patient's case. For smaller abscesses, antibiotics such as cephalosporins and aminoglycosides have proven effective [11]. In cases involving larger abscesses, these antibiotics are often used in conjunction with surgical procedures. For definitive treatment, open incision and drainage have demonstrated good results, while needle aspiration is also an option. Additionally, although endoscopic transurethral drainage has been tried, it has generally been less successful compared to other methods [9,12].

This case posed a significant clinical dilemma as the initial presentation was felt to be entirely acute and benign in nature. This was thought to be the case as generally abscesses develop acutely and are assumed to be infective in origin, as previously mentioned. Furthermore, the apparent improvement in the clinical picture following the initial treatment furthered this line of thinking. The case could represent two entirely independent pathologies, namely PGCC and a periurethral abscess. However, one may postulate that the aggressive PGCC may have contributed to tissue necrosis and fistula formation, predisposing the patient to abscess development. The aggressive nature of this variant is reflected in its rapid progression, poor differentiation, and high metastatic potential. Similar to previously reported studies, the patient in the case was elderly, had received previous treatment for PCa, and had an aggressive disease with rapid regrowth [4]. It is paramount to have a high index of suspicion in patients with abnormal presentations such as rapidly recurring periurethral abscesses and consider PGCC, especially in a patient with previous PCa. Further suspicions should arise when significant necrotic tissue is found at the time of drainage of a periurethral abscess in such a patient population. Early recognition will allow for appropriate and timely diagnosis and subsequent treatment.

## Conclusions

This case underscores the importance of having a high degree of suspicion of underlying malignant pathologies in patients presenting with acute illnesses. Furthermore, patients with a background of malignant disease should have early and in-depth investigations to ensure thorough examination. Though PGCC is a rare variant of PCa, there is established literature citing its development in those patients with previous PCa. It should be included in the differential diagnosis where appropriate. Where those patients with a background of PCa are undergoing acute intervention such as incising and draining abscesses, it may be prudent to send tissue not only for culture and sensitivity but also for histological diagnosis.
